# DeepATsers: a deep learning framework for one-pot SERS biosensor to detect SARS-CoV-2 virus

**DOI:** 10.1038/s41598-025-96557-8

**Published:** 2025-04-10

**Authors:** Ankhbayar Nyamdavaa, Kiran Kaladharan, Erdene-Ochir Ganbold, Seungdo Jeong, Seonuck Paek, Yansen Su, Fan-Gang Tseng, Tseren-Onolt Ishdorj

**Affiliations:** 1 Department of Computer Science, Mongolian University of Science and Technology, Ulaanbaatar, Mongolia; 2 Department of Engineering and System Science, National Tsing Hua University, Taipei, Taiwan, ROC; 3 Department of Physics, National University of Mongolia, Ulaanbaatar, Mongolia; 4 Department of Smart Information and Telecommunication Engineering, SangMyung University, Cheonan, Republic of Korea; 5 School of Artificial Intelligence, Anhui University, Hefei, 230601 China; 6 Department of Computer Science, New Mongol Institute of Technology, Ulaanbaatar, Mongolia; 7 Department of Software, SangMyung University, Cheonan, Republic of Korea

**Keywords:** Deep learning, CNN, GAN, Dataset augmentation, SERS, SARS-CoV-2 proteins, Biomedical engineering, Computational biology and bioinformatics, Computational platforms and environments, Data processing, Machine learning, Viral infection

## Abstract

The integration of Artificial Intelligence (AI) techniques with medical kits has revolutionized disease diagnosis, enabling rapid and accurate identification of various conditions. We developed a novel deep learning model, namely DeepATsers based on a combination of CNN and GAN to employ a one-pot SERS biosensor to rapidly detect COVID-19 infection. The model accurately identifies each SARS-CoV-2 protein (S protein, N protein, VLP protein, Streptavidin protein, and blank signal) from its experimental fingerprint-like spectral data introduced in this study. Several augmentation techniques such as EMSA, Gaussian-noise, GAN, and K-fold cross-validation, and their combinations were utilized for the SERS spectral dataset generalization and prevented model overfitting. The original experimental dataset of 126 spectra was augmented to 780 spectra that resembled the original set by using GAN with a low KL divergence value of 0.02. This significantly improves the average accuracy of protein classification from 0.6000 to 0.9750. The deep learning model deployed optimal hyperparameters and outperformed in most measurements comparing supervised machine learning methods such as RF, GBM, SVM, and KNN, both with and without augmented spectral datasets. For model training, a whole range of spectra wavenumbers ($320 \hbox { cm}^{-1}$ to $1650 \hbox { cm}^{-1}$) as well as wavenumbers ($1078 \hbox { cm}^{-1}$ and $1582 \hbox { cm}^{-1}$) only for fingerprint peak spectra were employed. The former led to highly accurate 0.9750 predictions in comparison to 0.4318 for the latter one. Finally, independent experimental spectra of SARS-CoV-2 Omicron variant were used in the model verification. Thus, DeepATsers can be considered a robust, generalized, and generative deep learning framework for 1D SERS spectral datasets of SARS-CoV-2.

## Introduction

The novel coronavirus (COVID-19), also known as Severe Acute Respiratory Syndrome Coronavirus 2 (SARS-CoV-2), is a severe acute respiratory syndrome that has spread around the world, about 236,533,988 people were affected by this virus^1^. The standard method for diagnosing COVID-19 is the Reverse Transcriptase Polymerase Chain Reaction (RT-PCR) test. But its time consuming and labor-intensive detection limits its effectiveness^2^. Lateral flow immunoassay (LFA), on the other hand, can be conducted on the spot by a layman within $\sim$15 min. However, it has inferior sensitivity compared to the PCR test^3^. Having other non-invasive automated diagnostic methods with high sensitivity and a rapid functioning diagnostic assay would be advantageous. In this regard, surface-enhanced Raman scattering (SERS) based immunoassay to detect SARS-CoV-2 has been an emergent technology developed lately. SERS has become one of the most widely used techniques for characterization and detection benefiting from its ultra-high sensitivity and molecule-specific spectrum. Nano-sized metal particles such as silver and gold greatly enhance the intensity of inelastic scattering of light by molecules up to factors of $\sim 10^8$, which is sufficient for single molecule detection in some cases^4–7^. In addition, relatively easy sample preparation, fast data collection, and the ability to investigate various types of samples with almost no damage have made Raman spectroscopy a unique and powerful tool for quantitative and qualitative analytical applications^8,9^. A variety of SERS biosensors to detect biomolecules have been developed for different purposes^4,10,11^.

Given this, a novel *one-pot SERS biosensor* to detect SARS-CoV-2, without any washing process using a portable Raman spectrometer was developed^4^. The biosensor substrate was fabricated using a silver deposited, antibody (Ab) immobilized microstructured Digital Versatile Disc (DVD-R) integrated with Raman reporter labeled silver nanoparticle (AgNP) for double clamping effects. The generated spectra were measured by a portable Raman Spectroscopic Apparatus Rapid-785 (Phansco Co. Limited, Taiwan). A comparison study using SARS-CoV-2 N protein and the biosensor showcased a much better sensitivity (> 100 times) relative to the commercial antigen test kits. Recently, AI-assisted SERS biosensors have been developed to accurately detect and precisely identify chemical substances and biological molecules from complex mixtures by intelligently analyzing the complicated experimental spectra^12–14^.

In the present study, we propose for the first time a novel deep learning (DL) model *DeepATsers* to utilize the one-pot SERS biosensor^4^. The DeepATsers model was designed as a combination of a convolutional neural network (CNN) and a generative adversarial network (GAN) to detect COVID-19 infections accurately and rapidly based on fingerprint-like molecular SERS spectra of each SARS-CoV-2 protein. The experimental spectroscopic data of the one-pot SERS biosensor feed DeepATsers for training. Independent SERS spectra of SARS-CoV-2 Omicron variant were used to verify the model.

Machine learning (ML) and deep learning (DL) paradigms have been extensively used in many domains, e.g., bioinformatics, medical information processing, diagnosis, and robotics, among many others. Specifically, convolutional neural networks, which we employed here, are the most utilized DL network type and play an important role in biomedical spectral data processing for diagnostic tools^15–18^.

Due to the limited amount of experimental Raman datasets of the SARS-CoV-2 S-protein (Spike), N-protein (Nucleocapsid), VLP protein (virus-like-particle), Streptavidin protein, and Blank signal generated by the one-pot SERS biosensor^4^, some dataset augmentation techniques such as extended multiplicative signal augmentation (EMSA)^19^, generated Gaussian-distributed noise^20^ and a more complex algorithm using a generative adversarial network (GAN)^21–23^ were applied to the spectroscopic dataset. To get a balanced spectral dataset representing each SARS-CoV-2 protein, k-fold cross-validation was employed. In the end, GAN outperformed the others having the most resembled datasets from the original ones having low Kullback-Leibler (KL) divergence^24^.

The performances of the machine learning and deep learning prediction models were evaluated by the different metrics of a confusion matrix (CM), receiver operating characteristic curve (ROC), the area under an ROC curve (ROC-AUC), and others^17,25^. The proposed deep learning model DeepATsers deployed optimal hyperparameter settings^26,27^ and demonstrates superior performance compared to other ML prediction models.

The present paper is organized as follows. In “Methods” section, we introduce the methods we utilized in the study, including dataset preparation and augmentation, model performance evaluation, and the design of the proposed DeepATsers. Then, in section “Results”, we present the extensive performance results of the ML and DL models on both the original experimental and the augmented datasets. We discuss the corresponding binary and multi-class classification behaviors of the models. We compare the classifiers based on the results to learn from the dataset in the range of whole wavenumbers versus only specific wavenumbers for peak spectra. Independently obtained settings were used for model testing. Finally, we conclude the performance analysis results of the proposed DeepATsers deep learning model in comparison with other machine learning models. We also remarked on further extension possibilities.

## Methods

### One-pot SERS biosensor and spectral data acquisition

In this study, the spectral dataset utilized was established by using a Raman spectrometer measuring the fingerprint SERS spectra of each SARS-CoV-2 protein: S protein, N protein, VLP, Streptavidin and Blank signal in Phosphate-buffered saline (PBS) and also VLP in untreated saliva. A biosensor with a sandwich immunoassay structure along with a silver-covered substrate, which was developed in our previous study^4^, generated the Raman spectrum of each protein. All SERS spectra were recorded in the wavenumber range from $320 \hbox { cm}^{-1}$ to $1650 \hbox { cm}^{-1}$ under 785 nm excitation with a resolution of $<10 \hbox { cm}^{-1}$. The characeristic peaks of 4MBA, Raman tagging molecule, at $1078 \hbox { cm}^{-1}$ and $1582 \hbox { cm}^{-1}$, which represent in the plane-ring breathing mode $\nu$(C-S) and $\nu$(C-C) vibrations bonds, were used to detect the SARS-CoV-2 proteins^4^.

Then one-pot SERS biosenser generated spectral datasets of the proteins, in turn, which were augmented to feed a data hungry deep learning model DeepATsers. The DL model performance results in comparison with other ML models for binary and multi-class classifications of the viral proteins were studied and verified by independent samples.

**Fig. 1:**
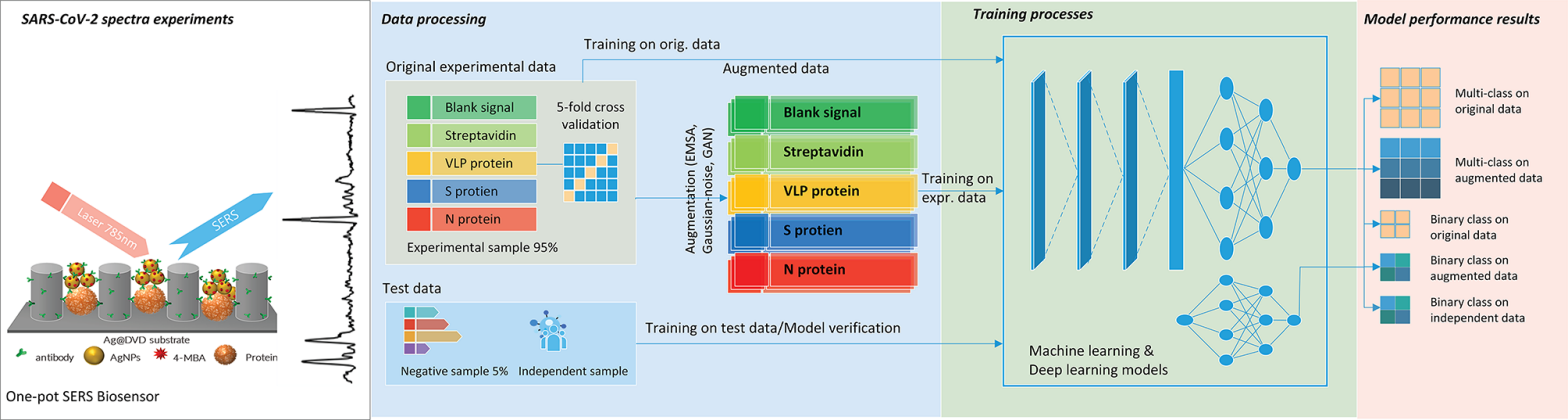
A schematic illustration of combined performances of a deep learning DeepATsers model with the one-pot SERS biosensor to identify the SARS-CoV-2 proteins based on their experimental Raman signatures. The SERS spectra were collected by a portable Raman spectrometer.

The schematic illustration of the study was presented in Figure 1.

### Dataset preparation and augmentation

We paid much attention to the dataset preparation to get a robust and effective model because models trained on a limited number of samples are often over-fitted, leading to inaccurate prediction results. Dataset augmentation generates synthetic data by transforming or learning from limited real data, preserving the class label effectively, and solving the constraints around a small number of experimental sample datasets^28–30^. The model should detect correct patterns from the data without producing too much noise. For this purpose, the k-fold cross-validation technique was used to prevent the models from overfitting and to ensure their performance on new, unseen data^31^. Cross-validation involves dividing the available data into multiple folds, using one of them as a validation fold, and the rest for training. This process is repeated multiple times, each time using a different fold as the validation set. Finally, the results from each validation step are averaged to get a more robust estimate of the model’s performance.

Because of the restricted number of SERS spectra of SARS-CoV-2 proteins obtained in^4^, augmentation techniques were used to expand the dataset: extended multiplicative signal augmentation (EMSA)^19^, generated Gaussian-distributed noise^20^ and a generative adversarial network (GAN)^21–23^. EMSA uses knowledge from the extracted background noise to suggest spectral augmentation by adding known captured noise variations. EMSA is based on the theoretical concepts underlying extended multiplicative signal correction (EMSC), a baseline correction algorithm applied to Raman spectral data. In the field of vibrational spectroscopy, EMSC has recently became one of the widely used technique for model-based preprocessing of molecular spectal data. It has received attention in quantitative analysis, molecular detection and surface analysis, and is used to remove unwanted variations from the measured spectra, like scattering features, interference fringes, fluorescence effects, or signal noise from the sample. Instead of simply making baseline corrections and removing unwanted variations, EMSC utilizes a mathematical model to identify and separate those effects to provide a more accurate correction^32,33^. In the Gaussian-noise model, random variations drawn from the same statistical distribution were added to the measured spectrum.

GAN is a class of deep learning models that consist of two neural networks, a generator *G*, and a discriminator *D* competing against each other. The generator’s role is to create synthetic data that resemble real data, while the discriminator’s job is to distinguish between real and generated data. The discriminator *D* provides feedback to generator *G* based on its predictions, guiding the generator to improve its output and create samples that closely resemble the original data. Meanwhile, the discriminator *D* aims to maximize the probability of correctly identifying whether a sample is real or generated. This adversarial process continues until the generator produces high-quality realistic data samples that are difficult for the discriminator to distinguish from the original data. The data can then be integrated into the training dataset. Indeed, it is a two-player min-max game as given in Eq. (1). A noise vector *z* from standard normal distribution is given as input to *G* which generates the *G*(*z*) as the output. Discriminator *D* classifies the input, it takes, as the real or generated data. It assigns a higher probability to real data that is if the input to the discriminator is *x* which is from real data distribution and a lower probability to the generated data that is if *x* is extracted from the generators data distribution over *x*, $p_g$.

1 $$\begin{aligned} min_Gmax_D~E_{x \sim p_{data}(x)}[log(D(x))]+E_{z\sim p_{g}(z)}[log(1-D(G(z)))] \end{aligned}$$

The loss function for the discriminator *D*:

2 $$\begin{aligned} L_D= E_{x \sim p_{data}(x)}[log(D(x))]+E_{z\sim p_{g}(z)}[log(1-D(G(z)))] \end{aligned}$$

The loss function of the generator *G*:

3 $$\begin{aligned} L_G= E_{z\sim p_{g}(z)}[log(1-D(G(z)))] \end{aligned}$$

We quantitatively measure the reflection of augmented spectrograms on real experimental spectra using Kullback-Leibler (KL) divergence^24^.

4 $$\begin{aligned} D_{KL}(R\parallel A) = \Sigma _{i=1}^NR(x_i)\cdot log\frac{R(x_i)}{A(x_i)}, \end{aligned}$$

where *A*(*x*) is the augmented dataset and *R*(*x*) is the real dataset. If these datasets match perfectly, $D_{KL}(R\parallel A)=0$ otherwise it can take values between 0 and $\infty$. The lower the KL divergence value, the better the matches between those two datasets *R* and *A*.

Initially, a total of 126 experimental datasets were provided in five groups of proteins and blank signals with different amounts (Section Methods). Six datasets of negative Streptavidin and blank signals were reserved for model testing. The remaining 120 were for training and validation. By applying each augmentation technique, the dataset of each group was extended to 156 and, in total, 780 spectral datasets were obtained in five groups. We implemented a five-fold cross-validation. The 780 spectral dataset was divided into 80% for training and 20% for validation. The results of each augmentation compared to the original spectra are shown in Fig. 3. One can see that the augmented dataset by Gaussian noise and EMSA resembles the original dataset well. However, GAN augmentation was the best way to fit the original dataset for the entire range of wave numbers, as shown in the second diagram in Fig. 3.

Now, both augmented and original (unaugmented) spectral datasets respectively feed the ML models of random forest (RF), gradient boosting machine (GBM), support vector machine (SVM), K-nearest neighbors (KNN), and a DL model of convolutional neural networks (CNN). The performance results of the models were illustrated in Fig. 4. GAN augmentation consistently demonstrated superior performance in both accuracy and F1 score for most models. In particular, CNN dominates with ACC=1.00 and F1 = 1.00 on the radar. This fact led to the design of a deep learning model based on a combination of CNN and GAN for accurate and rapid identification of viral proteins from its spectral datasets.

**Fig. 2:**
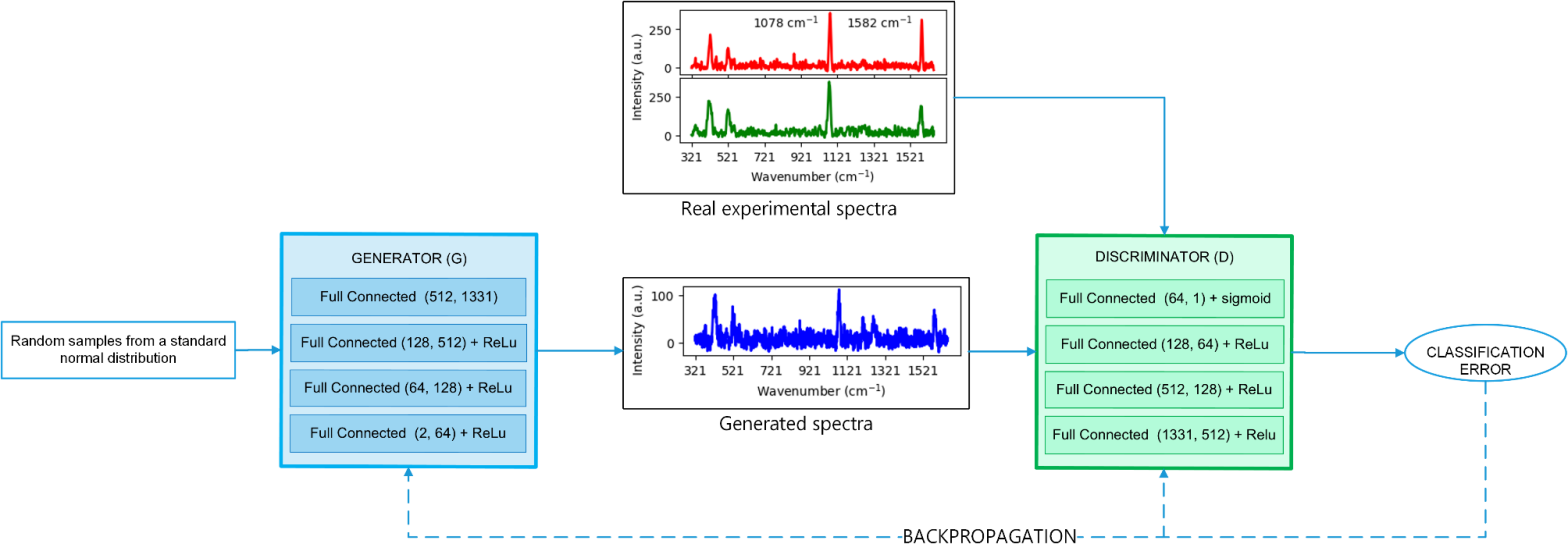
A schematic illustration of GAN based spectral dataset augmentation.

Here, we explain the GAN dataset augmentation strategies implemented in the current study (see Fig. 2). Firstly, to enhance the robustness of the discriminator, label smoothing was applied and assigned a value of 0.9 to the real experimental data. As a result, we got more generalized data than the original ones, which led to improved detection of the COVID-19 viral infection from the independent samples. Secondly, for further optimization of the training process, we ran the discriminator after every two to three iterations of the generator allowing it to learn and generate more realistic synthetic data, where a learning rate of 0.0002 was set for the discriminator and 0.0001 for the generator. Such an alternating training approach fosters a more balanced adversarial process, contributing to the effectiveness and stability of the model. To be clear, the spectral datasets with and without augmentation were compared in Figs. 5 and 6. Firstly, a 3D diagram depicts the spectrum of each viral protein and the blank signal measured during the experiment. Following that, 3D projections of those positive and negative samples and the multi-protein partition were plotted. These original datasets exhibit a more scattered distribution, making it challenging to differentiate between segments, likely due to the limited data size.

After an extensive training regimen spanning 10,000 epochs, the GAN augmentation demonstrated remarkable progress in its capacity to generate data that closely resembled the real experimental data. The model gradually refined its parameters and learned intricate patterns in the data distribution, ultimately achieving a level of fidelity where its generated outputs became almost indistinguishable from the real data. We see the results of iterative learning processes of 1,000, 4,000, 6,000, and 10,000 epochs and finally obtain a highly resembled form of the augmented dataset from the original S protein spectrum. Its indistinguishability is ensured by the quantitative values of the lower KL divergence (0.0206; 0.0214) presented in Table 1 and in Figure 6.

**Fig. 3:**
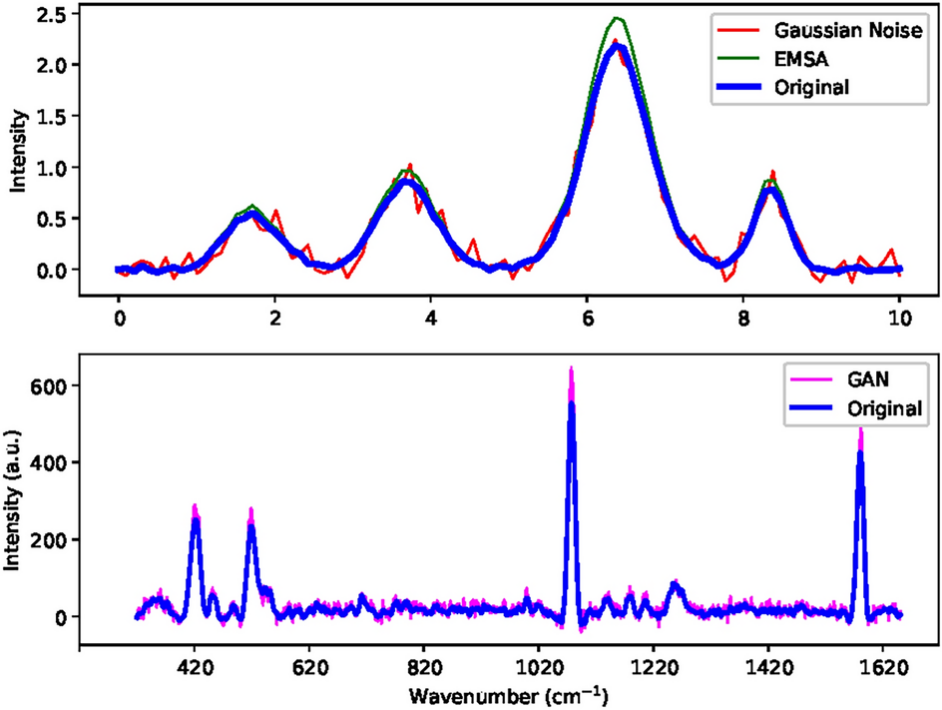
Comparison of the original spectra versus EMSA, Gaussian-noise, and a GAN-based augmented dataset.

**Fig. 4:**
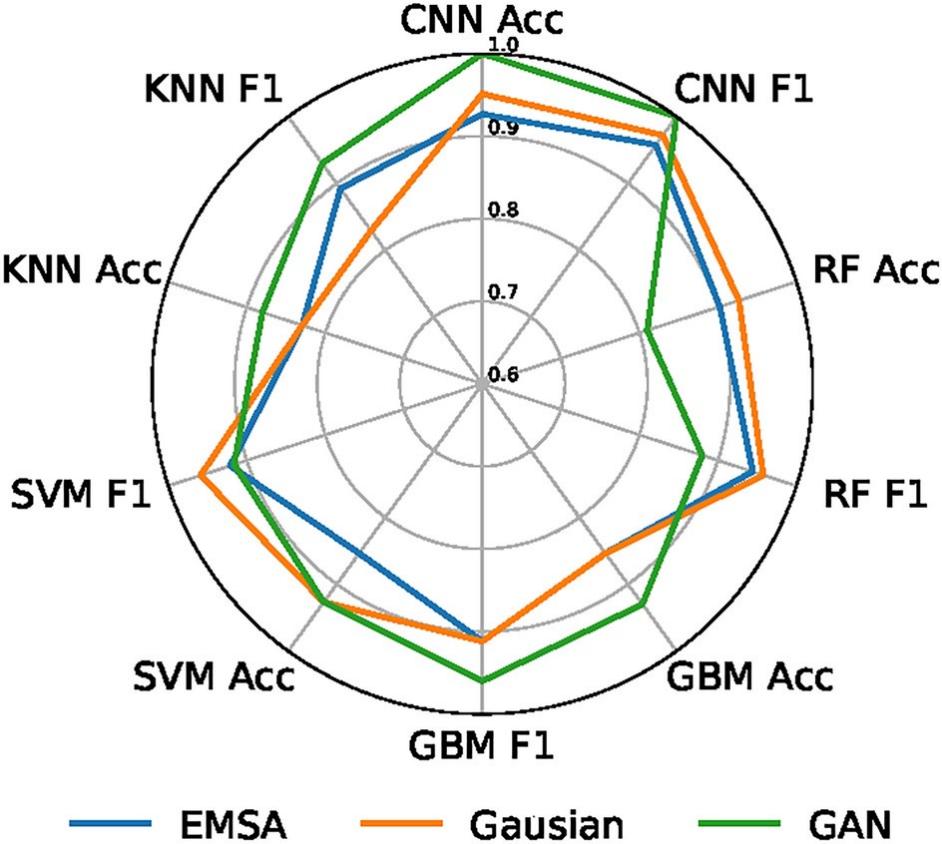
Performance comparison of RF, GBM, SVM, KNN, and CNN classifiers on Gaussian-noise, EMSA, and a GAN-based augmented dataset.

**Table 1:** KL divergence of real experimental and GAN augmented spectral datasets for different epochs.

Epochs	$D_{KL}(R\parallel A)$	$D_{KL}(A\parallel R)$
1,000	0.1977	0.1903
4,000	0.1449	0.1487
6,000	0.1262	0.1339
10,000	0.0206	0.0214

**Fig. 5:**
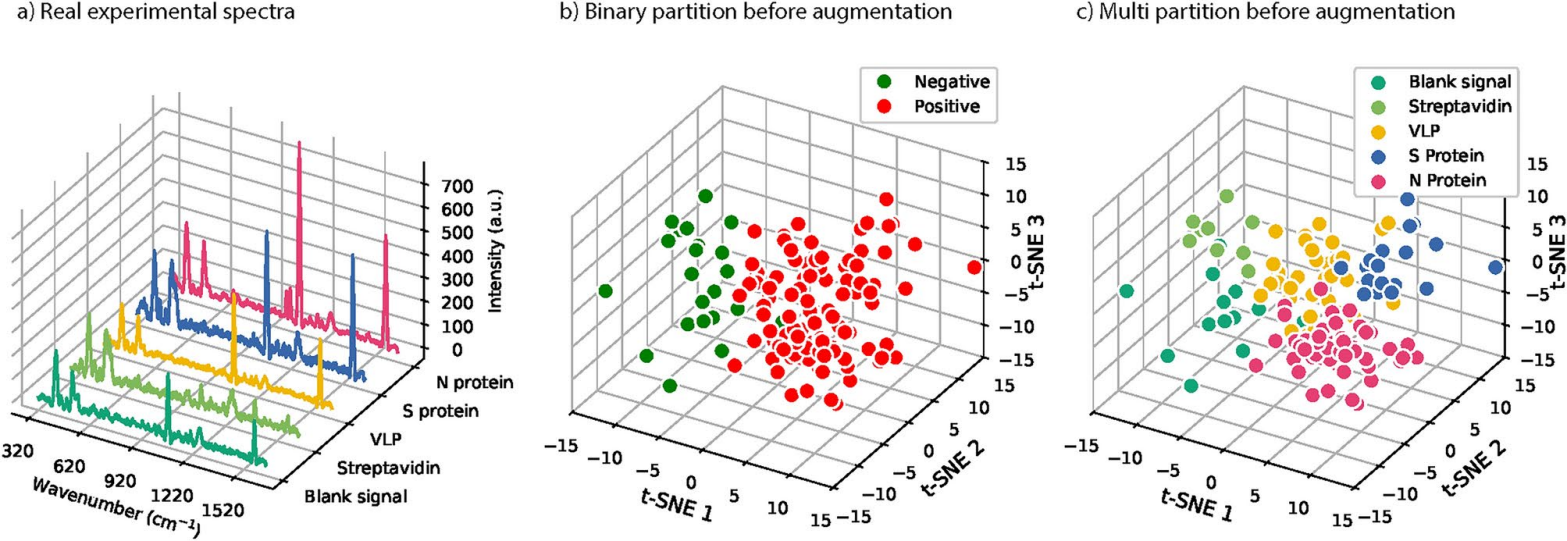
(**a**) SERS experimental spectra of SARS-CoV-2 proteins and the blank signal. (**b**) Binary partition of positive and negative samples. (**c**) Multi-protein partition was projected with t-SNE,^34^.

**Fig. 6:**
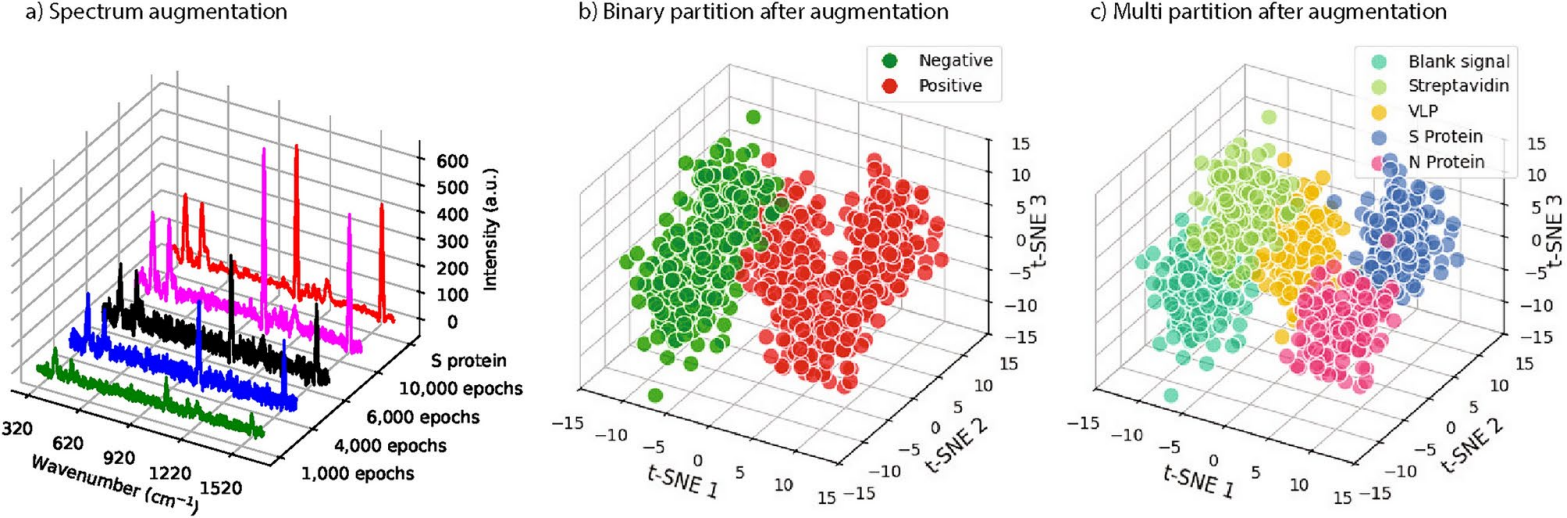
(**a**) GAN augmentation iterative process for the S protein spectrum. (**b**) Binary partition and (**c**) multi-partition of the augmented spectral datasets of the viral proteins were plotted, respectively, with t-SNE.

We recognize that we have achieved notably positive results of GAN augmentation for the 1D spectral dataset, despite its common utilization in 2D images^35,36^.

### Model performance evaluation

The classification models’ performance analysis relies on certain accuracy metrics, such as the confusion matrix (CM), the receiver operator characteristic (ROC), and the area under the curve (AUC).

The confusion matrix is a commonly used technique where each row represents an actual class and each column represents the state of a predicted class. In the confusion matrix, TP is the number of true positives if the prediction and actual value are both positive; FP is the number of false positives if the prediction value is positive and the actual value is negative; TN is the number of true negatives if the prediction and actual value are both negative; and FN is the number of false negatives if the prediction value is negative but the actual value is positive. The next formulas calculate the evaluation measurements (^17,25^):

$$\begin{aligned} {\small \begin{array}{lll} \texttt {ACC~(Accuracy)}=\frac{\texttt {TP+TN}}{\texttt {TP+TN+FP+FN}}, & \texttt {TPR~(Sensitivity)}=\frac{\texttt {TP}}{\texttt {TP+FN}},& \texttt {NPV~(Negative~pred.~value)}=\frac{\texttt {TN}}{\texttt {TN+FN}},\\ \\ \texttt {TNR~(Specificity)}=\frac{\texttt {TN}}{\texttt {TN+FP}},& \texttt {PPV~(Precision)}=\frac{\texttt {TP}}{\texttt {TP+FP}},& \texttt {f1-score}=\frac{\texttt {2*Sensitivity*Precision}}{\texttt {Sensitivity+Precision}}. \end{array}} \end{aligned}$$

*Accuracy* evaluates how close the result is to the measurement’s true value (positive and negative). *Sensitivity* tells us what proportion of the positive class got correctly classified. *Specificity* tells us what proportion of the negative class got correctly classified. *Positive predictive value* is a proportion of truly positive cases among the positive cases detected. *Negative predictive value* is about the proportion of truly negative cases among the negative instances detected. The f1-score tells us the predictive skill of a model by elaborating on its class-wise performance rather than an overall performance as done by accuracy.

The ROC is a two-dimensional graph used as a measurement tool for the performance evaluation of the classifiers where TPR is plotted on the *Y* axis while FPR is plotted on the *X* axis. An ROC graph depicts trade-offs between TP and FP: the point (0, 1) represents perfect classification, the point (0, 0) represents no positive classification issuing, and the point (1, 1) represents positive classification. The diagonal line $x=y$ represents the strategy of randomly guessing a class. The point (0.5, 0.5) in ROC space represents a half positive and half negative classification.

The area under the ROC curve (AUC-ROC) measures the ability of a binary classifier to distinguish between classes and is used as a summary of the ROC curve. The higher the AUC, the better the model’s performance in distinguishing between the positive and negative classes. An AUC score of 1 means the classifier can perfectly distinguish between all the positive and the negative class points while a value of 0 shows that the classifier predicts all negatives as positives and vice versa. AUC 0.5 says that the classifier is not working. A value above 0.5 means the classifier can detect more numbers of true positives and true negatives than false negatives and false positives.

### Deep learning model design

Deep learning models including CNNs have attracted much attention for biomedical modeling and applications. In particular, it has emerged for 1D Raman spectral data recognition and analysis of biomolecules^37^. Deep learning model architecture combines all the data preprocessing, feature extraction, and classification processes. It can be trained end-to-end without human intervention. This simplifies the development of a machine classification system and also achieves significantly higher accuracy for a variety of different biological samples using their Raman spectroscopic data^38–40^.

However, because of the difficulty of measuring the weak Raman spectra of nanoscale experimental samples and acquiring a sufficient amount of highly qualified spectroscopic data generated in the laboratory experiment^4^ to feed deep learning models, we designed a specific deep learning network architecture *DeepATsers*. The DeepATsers combines GAN data augmentation technique with a CNN training model. It was specifically designed for a 1D SERS spectral dataset of SARS-CoV-2 proteins to identify each protein in certain media with a high sensitivity and specificity based on their intrinsic fingerprint-like molecular spectrum.

**Fig. 7:**
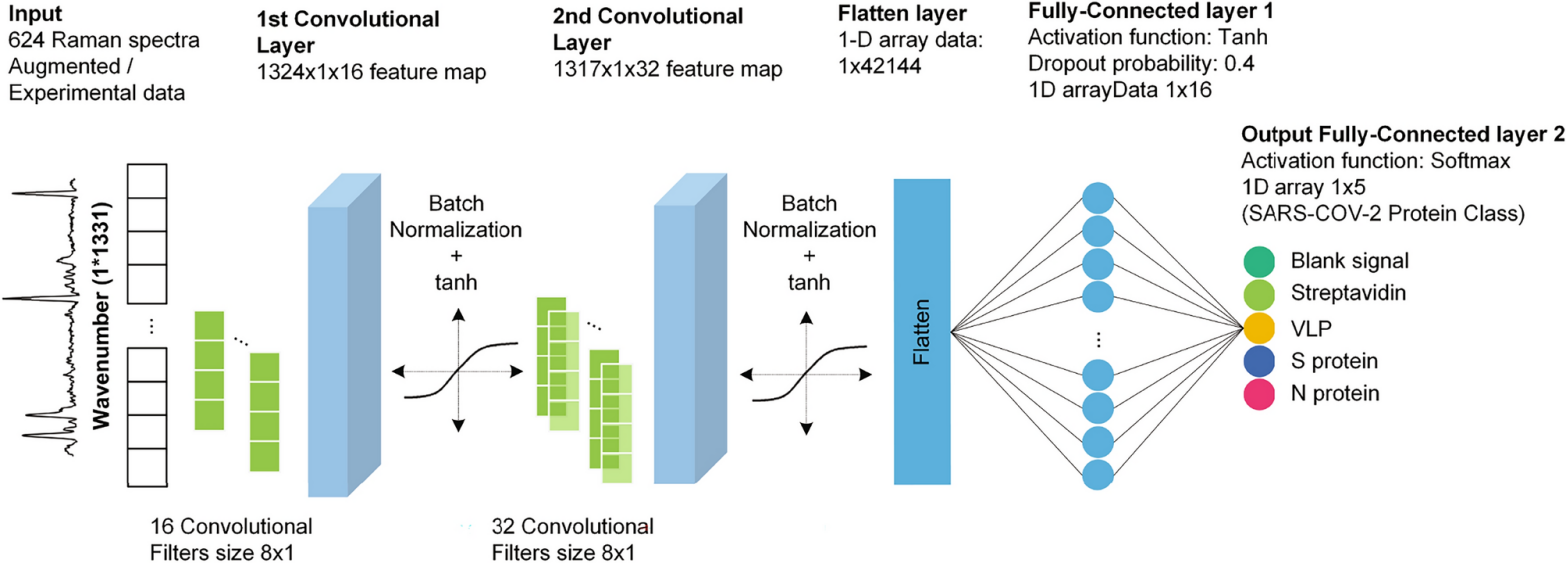
Architecture of the DeepATsers deep learning model.

The entire architecture of DeepATsers was composed of 6 layers: 2 convolutional layers, 1 dropout layer, 1 flatten layer, and 2 fully connected layers, see Figure 7.

The 624 pieces 1D SERS spectra of size 1$\times$1331 for each feed the deep learning model for training. We explain each layer of the architecture along with hyperparameters as follows. The 1st layer of convolutional 1D (Conv1D) has 16 filters to learn from the input. The 2nd layer uses 32 filters. At these layers, convolution is performed by shifting the kernel across the input SERS dataset with a stride of 1 through the input from the beginning to the end. As a result, the first layer is composed of a feature map with 1324$\times$1$\times$16 size while the second layer has a feature map of 1317$\times$1$\times$32. After each convolutional layer, tanh activation function (Eq. 5) utilizes the batch normalization output.

5 $$\begin{aligned} \texttt {tanh}(x) = \frac{e^{x} - e^{-x}}{e^{x} + e^{-x}}, \end{aligned}$$

where *x* can be a real number representing the input or the output from the previous layer, and a weighted sum of inputs after adding a bias term. The tanh function outputs values in the range $-1$ to 1 and produces more pronounced gradients. This characteristic can facilitate faster learning and convergence during training, making it more resistant to issues related to vanishing gradients.

By applying batch normalization after the convolutional layers, the model addresses internal covariate shifts, maintaining consistent gradient distributions during training. This approach improves robustness while introducing slight noise from mini-batch statistics, serving as a regularization technique to combat overfitting. The integration of batch normalization into our DeepATsers model significantly enhances the learning capability, allowing it to effectively capture complex patterns present in the SERS spectral data of the SARS-CoV-2 proteins.

In the flatten layer, all activated features are concatenated and flattened into a 1D array of 1$\times$42,144 spectra. Consequently, the first fully connected layer of size 1$\times$16 was built by the activation function of tanh and applying a dropout probability of 0.4 to prevent overfitting. Finally, the Softmax activation function (Eq. 6) was applied to the output layer to obtain a reliable classification.

6 $$\begin{aligned} \sigma (\overrightarrow{z})_i=\frac{e^{z_i}}{\Sigma _{j=1}^Ke^{z_j}}, \end{aligned}$$

where all the $z_i$ values are the elements of the input vector and can take any real value. The denominator of the formula is the normalization term, which ensures that all the output values of the function will sum to 1, thus constituting a valid probability distribution. The output layer consists of a 1$\times$5 array. The whole model does multi-class classification for the 5 types of SARS-CoV-2 proteins. It also can do binary classification by only replacing the output layer as a 1$\times$2 array to predict whether the sample is positive or negative. The model was compiled using the Adam optimizer, known for its adaptive learning rates, and the loss function was sparse_categorical_crossentropy (SCCE), see Eq. (7), suitable for multi-class classification tasks to measure the dissimilarity between the prediction *Y* and the actual outcome $\hat{Y}$.

7 $$\begin{aligned} \rho (Y,\hat{Y})=-\frac{1}{n}\Sigma _{i=1}^nlog(\hat{y}_{i,y_i}), \end{aligned}$$

where $\hat{y}_i$ is the integer label as an index for the true class of the *i*-th sample, and $\hat{y}_{i,y_i}$ is the predicted output for the *i*-th sample and true class label. The classes are encoded as integers rather than one-hot encoded vectors.

Accuracy was used as a performance metric. The training was carried out with a batch size of 32 across 100 epochs, allowing the model to iteratively adjust its weights for an improved classification of the Raman spectral data. The learnable parameters updated during the training and the optimal settings of crucial hyperparameter values obtained by manual tuning (see Table 3) were presented in Table 2. These were suitable in the DeepATsers model for both binary and multi-class classifications at the same time. We also checked the hyperparameter values obtained by automatic search such as Grid search tuning^27^. It was good for binary classification but not good for multi-class classification, having low accuracy of 0.71. This led us to omit it. For more information on Grid tuning results, see Supplementary Table S3 and Supplementary Figure S7 and S8.

**Table 2:** Estimated parameters and hyperparameter setup of DeepATsers model. Weights shape (x,y,z) where x,y—kernel size, z—# of filters. A/B—A for binary classification, B for multi-class (5) classification.

Parameters	Values	Hyperparameters	Values
1st layer weights shape	(8, 1, 16)	Hidden layers	2
(weights, bias)	(128, 16)	Kernel size	8
Trainable parameters	144	Activation functions	tanh, Softmax
Batch normalization	64	Stride	1
2nd layer weights shape	(8, 16, 32)	Epochs	100
(weights, bias)	(256, 32)	Drop out rate	0.4
Trainable parameters	4,128	Learning rate	0.001
Batch normalization	128	Optimizer	Adam
Full connected layer weights shape	(42144, 16)	Batch size	32
(weights, bias)	(674304, 16)	Loss function	SCCE
Trainable parameters	674,320	Input size	1$\times$1,331
Output layer weights shape	(16, 2/5)	Weight initialization	Xavier
(weights, bias)	(32, 2/5)	Dropout layer	1
Trainable parameters	34/85	Flatten layer	1
Total trainable parameters	678,722/678,773	Full connected layer	2
Total optimizable parameters	1,357,446/1,357,548	Feature maps	16 and 32

**Table 3:** Hyperparameter tuning results.

Layers	Accuracy	Kernel	Accuracy	Dropout rate	Accuracy
1	0.9286	6	0.9643	0.0	0.8924
**2**	**0.9643**	**8**	**0.9643**	0.2	0.9643
3	0.8571	16	0.8929	**0.4**	**0.9643**
4	0.7857	32	0.8929	0.6	0.9286
5	0.7857	48	0.8937	0.8	0.8929

## Results and discussion

### Model performance analysis

The dataset preparation technique was explained in Section “Dataset preparation and augmentation”. The GAN augmented $780\times 1331$ SERS spectral dataset of SARS-CoV-2 proteins, split into 80% ($624\times 1331$) for training and 20% ($156\times 1331$) for validation, was also deployed in the DeepATsers model and some well-known machine learning models RF, GBM, SVM and KNN as well. The parameters and hyperparameters of the ML models, respectively, were represented as shown in Table 4.

**Table 4:** Parameters and Hyperparameter settings of ML models.

Models	Parameters	Hyperparameters
RF	Average tree depth: 7.94	n$\_$estimators: 300, max$\_$depth: 300, min$\_$samples$\_$leaf: 1
GBM	Average tree depth: 10.93	n$\_$estimators: 500, max$\_$depth: 300, learning$\_$rate: 0.05, random$\_$state: 100, max$\_$features: 5
SVM	Number of Support Vectors for each class: [89 86], Bias(b): 0.15887355	kernel: rbf, c: 2.0, gamma: scale
KNN		n$\_$neighbors: 2, metric: minkowski, weights: uniform

Because detecting viral protein presence is crucial and the imbalanced objects appeared in the positive (65%) and negative (35%) classes, the binary classification threshold was set to 0.2 based on the *precision-sensitivity metrics trade-off*. This yields a higher sensitivity score for each model when binary class classifications were carried out.

**Fig. 8:**
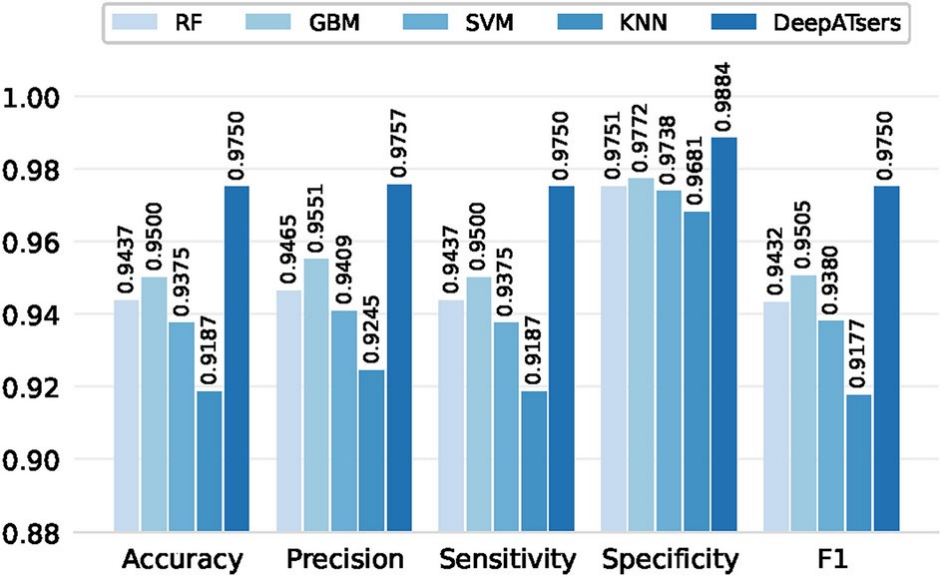
Multivariate ML and DL model performance metrics on the augmented datasets in Table 8. DeepATsers dominates.

The prediction results of DeepATsers and other machine learning models on the SERS datasets of SARS-CoV-2 proteins and baseline with and without augmentation are presented in Table 5. This shows that ML and DL models reach from 0.01 to 0.1 higher scores for the highest prediction metrics on the augmented dataset in comparison to the original experimental dataset. Moreover, DeepATsers outperforms other ML models on augmented datasets according to the evaluation metrics of accuracy (0.9575), sensitivity (0.9575), and specificity (0.9901) (see Fig. 8).

**Fig. 9:**
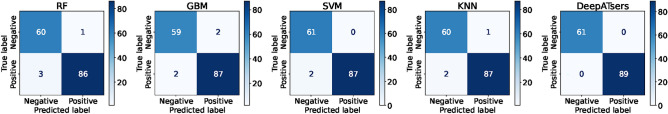
Confusion matrices of ML and DL models for binary classification of SARS-CoV-2 proteins on the augmented spectral datasets.

In Fig. 9, the confusion matrices of each ML and DL model express excellent prediction results: 97% for RF, 97% for GBM, 98% for SVM, 98% for KNN, and 100% for DeepATsers.

An important note is that all training processes to identify viral proteins based on their fingerprint-like spectra were performed using a whole range of wavenumbers from $320 \hbox { cm}^{-1}$ to $1650 \hbox { cm}^{-1}$, and the two specific wavenumbers of $1078 \hbox { cm}^{-1}$ and $1582 \hbox { cm}^{-1}$ only for SERS spectra peaks of the SARS-CoV-2 protein signatures. In the former case, the models learn from an extensive spectral dataset and get more accurate results, while in the latter case, a restricted dataset is provided for the models to learn. As a result, we got a surprising result that the training on the whole wavenumber range gives a much higher accuracy of 0.9242 compared to training on the two wavenumbers only, which has an accuracy of 0.4924. The detailed comparisons of each model result for the wavenumbers range versus the two wavenumbers alone are presented in Table 6. The results indicate that training on the full spectrum wave range is better at predicting the individual components in complex mixtures. This is likely due to the additional information contained in the full spectrum, which allows the machine learning algorithm to better distinguish between the subtle differences in the spectral signatures of the various components.

**Table 5:** ML and DL model binary classification analysis of SARS-CoV-2 proteins with and without augmented experimental datasets, respectively.

Model	Accuracy		Precision		Sensitivity		Specificity		f1-score	
	Real data	Augm. data	Real data	Augm. data	Real data	Augm. data	Real data	Augm. data	Real data	Augm. data
RF	0.9231	0.9733	0.9524	0.9885	0.9524	0.9663	0.8000	0.9836	0.9524	0.9773
GBM	0.8846	0.9733	0.8750	0.9775	1.0000	0.9775	0.4000	0.9672	0.7000	0.9775
SVM	0.8462	0.9867	0.8696	1.0000	0.9524	0.9775	0.4000	1.0000	0.9091	0.9886
KNN	0.8846	0.9800	0.9500	0.9886	0.9048	0.9775	0.8000	0.9836	0.9268	0.9831
DeepATsers	0.8462	1.0000	0.8696	1.0000	0.9524	1.0000	0.4000	1.0000	0.9091	1.0000

**Table 6:** ML and DL model performance accuracies for multi-class, binary classification on both with and without augmented experimental datasets for whole spectral wave range and two wavenumbers for peaks only. The same for binary classification on independent samples.

Model	Multi-class classification		Binary classification		For independent sample	
	Waves for 2 peaks	Wave range	Waves for 2 peaks	Wave range	Waves for 2 peaks	Wave range
RF	0.6439	0.9437	0.9470	0.9733	0.8571	0.9286
GBM	0.5909	0.9500	0.9015	0.9733	0.8095	0.9524
SVM	0.6288	0.9375	0.8864	0.9867	0.5000	0.6429
KNN	0.6667	0.9187	0.9545	0.9800	0.7857	0.6667
DeepATsers	0.4318	0.9750	0.7652	1.0000	0.6667	0.9643

### Model verification

**Fig. 10:**
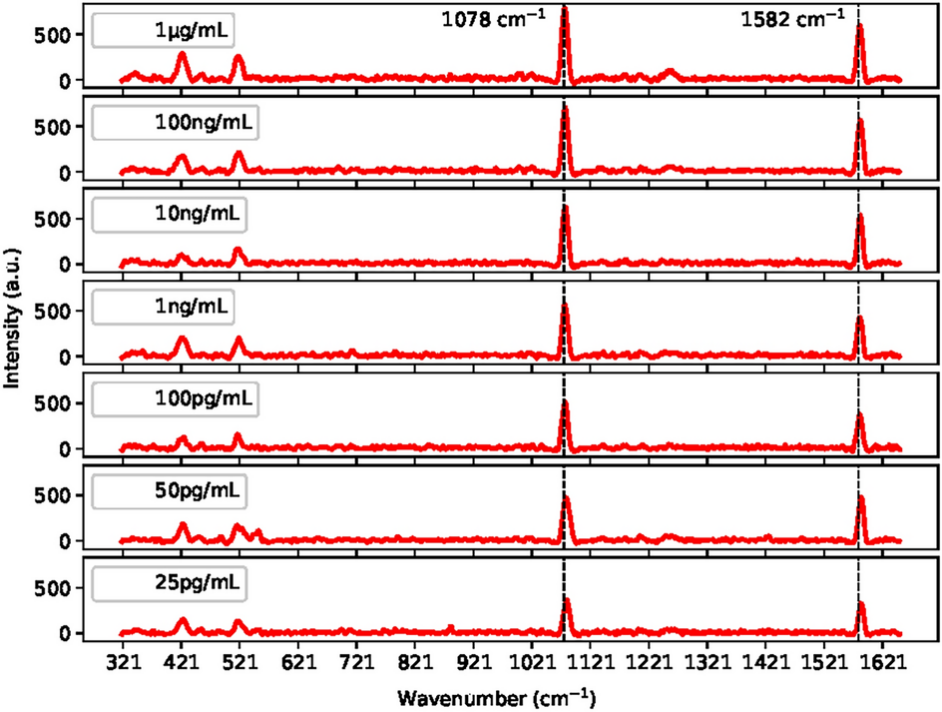
SERS spectra of SARS-CoV-2 Omicron obtained from six different concentrations.

**Fig. 11:**
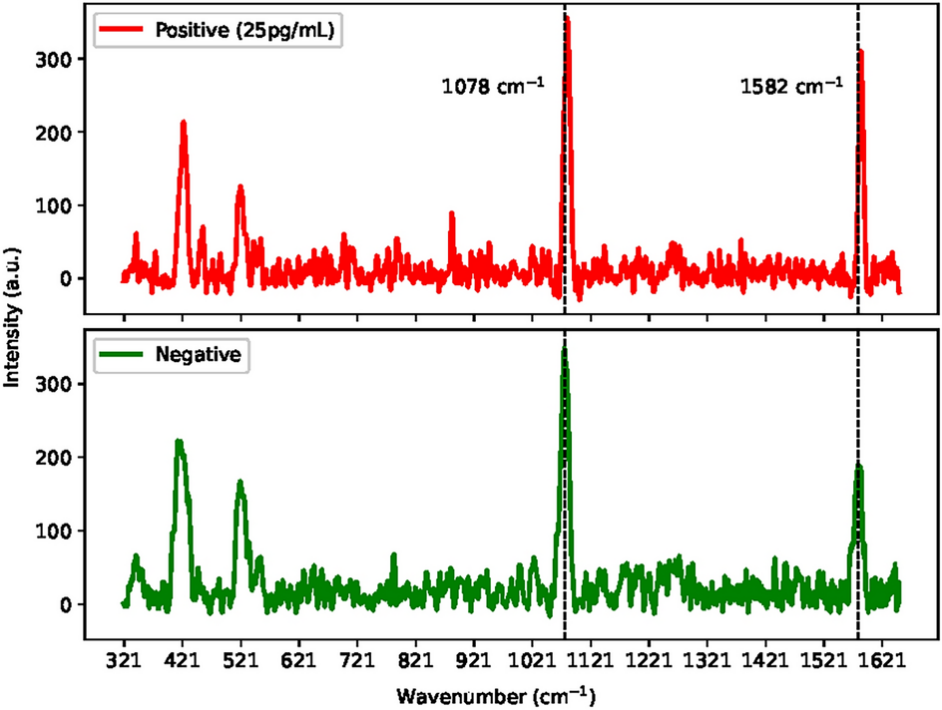
SERS spectrum of an independent sample of 25 pg/mL Omicron variant (above). SERS spectra of the negative samples (below).

The performance of the DeepATsers model was verified using a batch of test datasets comprising 6 reserved experimental negative samples, recall Dataset preparation and augmentation section, and 22 independent (not used in model training) positive spectral datasets. The independent sample was collected from seven different concentrations (1 $\upmu$g/mL, 100 ng/mL, 10 ng/mL, 1 ng/mL, 100 pg/mL, 25 pg/mL) of the SARS-CoV-2 Omicron variant as illustrated in Fig. 10. The six reserved negative experimental samples are also shown in Fig. 11 with a comparison of the 25 pg/mL sample. The independent and experimental samples have very similar characteristics, which shows the highly qualified datasets we got for modeling.

**Table 7:** Model verification results using with and without augmented independent samples.

Model	Accuracy	Precision	Sensitivity	Specificity	f1-score	ROC-AUC
Trained on experimental data	0.7857	0.9000	0.8182	0.6667	0.8571	0.7424
Trained on augmented data	0.9643	0.9565	1.0000	0.8333	0.9778	0.9167

**Fig. 12:**
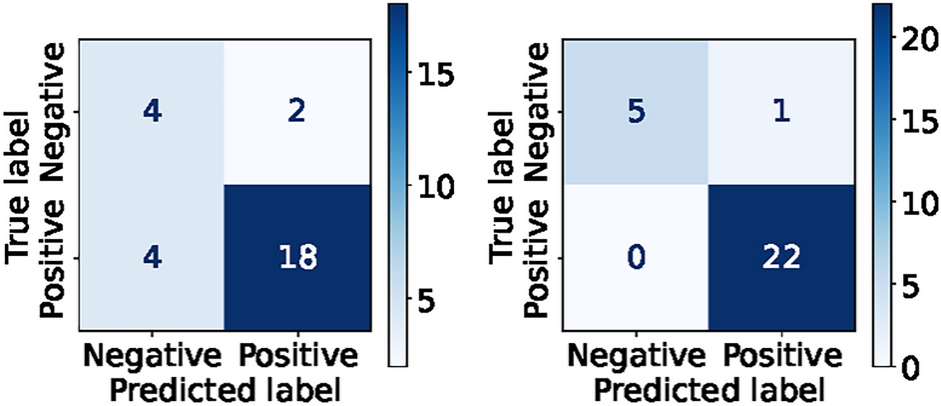
Model verification: $\sim$25% error rate without augmentation (left); $\sim$96% correct prediction on augmented dataset (right).

The DeepATsers model was trained on both with and without augmented SERS spectral datasets achieving accuracies of 0.7857 and 0.9643 and sensitivities of 0.8182 and 1.0000, respectively, as presented in Table 7. The high sensitivity of 1.00 of the model performance says that diagnostic capability remains robust even in the face of input dataset change. The proposed deep learning model demonstrated exceptional performance on the independent sample datasets as the results presented in Table 7 and the confusion matrix in Fig. 12 show, achieving 96% correct classification out of 28 SERS-CoV-2 antigen spectra. It indicates that DeepATsers has a strong ability to utilize the one-pot SERS biosensor for training, validation, and testing.

### Identification of each SARS-CoV-2 protein

In this section, we learn about the performance of the DeepATsers model to predict each SARS-CoV-2 protein of N protein, S protein, VLP protein, Streptavidin, and Blank signal in a media of PBS and VLP protein in saliva.

**Fig. 13:**
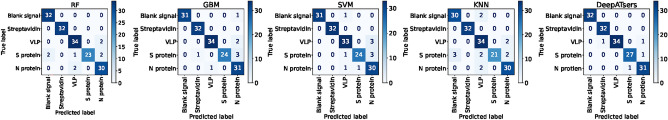
Confusion matrices for multi-class classification of SARS-CoV-2 proteins.

Table 8 presents the multi-class classification results for each SARS-CoV-2 protein and blank signal. Consider that the ML and DL models were deployed. Note that the performance metrics of each model were built as weighted averages of corresponding classes. For the real experimental dataset, the performance metrics of each ML and DL model fall in the range from 0.3404 to 0.8520, which is not satisfying. While for the augmented dataset, all models present excellent scores above 0.9177 (see Table 8). One can observe from the table that the CNN-based DeepATsers model outperforms ML models in all metrics having scores from 0.9618 to 0.9901 on the augmented datasets. Multi-class classification confusion matrices of each ML and DL model for each viral protein are depicted in Fig. 13. We can explore detailed prediction results of the models for viral protein classifications. For instance, the worst prediction was by KNN having 13 wrong while the best was DeepATsers having only 6 errors.

Multi-class classification for the proteins on both with and without augmented experimental datasets was performed, and the accuracies are presented in Table 9. Despite a few prediction errors that occurred with VLP protein in PBS and in saliva as in the last confusion matrix of DeepATsers of Fig. 13, it has an excellent prediction ability of more than 90% accuracy for each protein.

**Table 8:** ML and DL multi-class classification results for SARS-CoV-2 proteins using the real experimental dataset and the GAN augmented dataset, respectively.

Model	Accuracy		Precision		Sensitivity		Specificity		f1-score	
	Real data	Augm. data	Real data	Augm. data	Real data	Augm. data	Real data	Augm. data	Real data	Augm. data
RF	0.5600	0.9437	0.4933	0.9465	0.5600	0.9437	0.7904	0.9851	0.5040	0.9432
GBM	0.5200	0.9500	0.3404	0.9551	0.5200	0.9500	0.7904	0.9872	0.4095	0.9505
SVM	0.6000	0.9375	0.4745	0.9409	0.6000	0.9375	0.8281	0.9838	0.5268	0.9380
KNN	0.6400	0.9187	0.5806	0.9245	0.6400	0.9187	0.8520	0.9781	0.6038	0.9177
DeepATsers	0.6000	0.9750	0.5529	0.9757	0.6000	0.9750	0.7594	0.9984	0.5479	0.9750

**Table 9:** Multi-class classification results of SARS-CoV-2 proteins by DeepATsers using both with and without augmented datasets.

Model	ACC on real sample	ACC on augm. data
Blank signal	0.6923	1.0000
Streptavidin	0.7692	1.0000
VLP	0.6923	0.9666
S protein	0.6154	0.9047
N protein	0.6154	0.9705

**Fig. 14:**
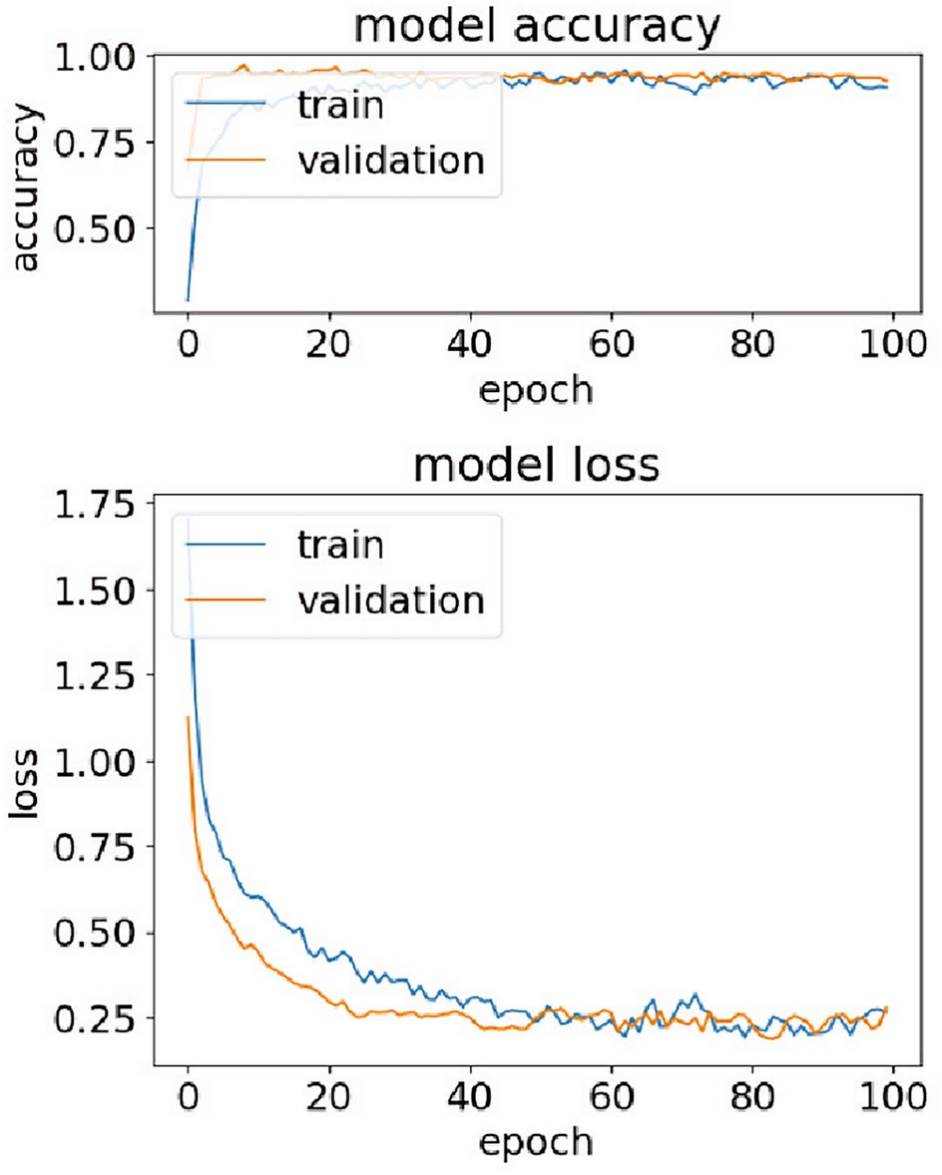
DeepATsers performance accuracy and loss over 100 epochs.

**Fig. 15:**
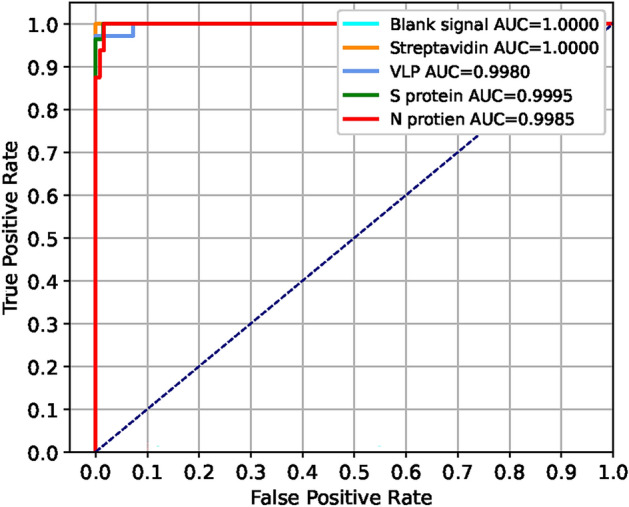
ROC of DeepATsers multi-class classification, according to Table 9.

Considering the capability of the DeepATsers model, the accuracy-epoch curve and loss-epoch curve depicted in Fig. 14 plots a high accuracy value of 0.9930 and low loss value of 0.02 for each training and validation dataset over 100 epochs, showing no overfitting. In the multi-class classification of the viral proteins, the One-versus-Rest approach was applied to compute ROC-AUC and got an average score of 0.9992 as depicted in Fig. 15. These quantitative facts prove that the DeepATsers has a great ability to identify each SARS-CoV-2 protein from its SERS spectral characteristics.

## Conclusions

We proposed a deep learning model DeepATsers to utilize a novel one-pot SERS biosensor^4^ to identify the COVID-19 infection rapidly. The DeepATsers design combines CNN with GAN to expand the limited number of experimental SERS spectra and then make predictions. The SERS datasets of the five groups of SARS-CoV-2 proteins and the blank signal were generalized using a 5-fold cross-validation to prevent overfitting. The GAN augmentation highly resembled the original 1D spectral datasets providing a low KL divergence value of 0.02. This is better in comparison to EMSA and Gaussian noise. Then the synthetic spectral dataset significantly enhanced the training process. The training process also examined the full spectral wavenumber range in contrast to focusing solely on the wavenumbers for the peaks. This strategy reduces the influence of small variations in fingerprint spectra that could be caused by the toxicity of silver nanoparticles^41^.

We evaluated the capabilities of commonly used supervised machine learning methods against the DeepATsers to analyze SERS spectra. In comparative experiments conducted with the augmented datasets, models such as RF, GBM, SVM, KNN yielded average accuracies of 0.9437, 0.9500, 0.9375, 0.9187, respectively, while DeepATsers was highest 0.9750. Note that manually tuned optimal hyperparameters set up the DeepATsers for both binary and multi-class classifications rather than automatic Grid search tuning results since the latter one does not fit for the classifications at the same time. The trainable parameters of the ML and DL models, respectively, were presented.

Finally, DeepATsers was well verified by using independent SERS spectra of SARS-CoV-2 antigens and reserved negative experimental samples as well.

Thus, the excellent performance results of the DeepATsers model suggest that the robust approach not only enhanced the detection ability but also ensured high accuracy and possible reliability in real-world diagnostic scenarios. We have shown that leveraging the strengths of deep learning and the one-pot SERS biosensor can significantly improve diagnostic capabilities, which is essential for timely pandemic responses.

Furthermore, DeepATsers’ approach holds great promise for identifying SARS-CoV-2 variants, thus enhancing our ability to monitor and respond to the virus’s evolving strains. It is possible to extend DeepATsers by combining it with the Gaussian-Lorentzian^42^ function to identify crucial fingerprint peaks of individual biological and chemical entities. This could make the model more general and allow the detection of multiple entities simultaneously in the one-pot mixed sample without prior knowledge.

## Electronic supplementary material

Below is the link to the electronic supplementary material.

Supplementary Information.

## Data Availability

All data generated or analysed during this study are included in this published article and its supplementary information files.
