# Supplementary material for: DeepATsers: a deep learning framework for one-pot SERS biosensor to detect SARS-CoV-2 virus
Source: Sci Rep. 2025 Apr 10;15:12245. doi: 10.1038/s41598-025-96557-8 (PMC11985927; doi:10.1038/s41598-025-96557-8)
Supplement: Supplementary file 1 — Supplementary Information. [file 41598_2025_96557_MOESM1_ESM.pdf]

Supplementary of DeepATsers: A Deep learning framework for one-pot SERS biosensor to detect SARS-CoV-2 virus

Ankhbayar Nyamdavaa[1,6], Kiran Kaladharan[2], Erdene-Ochir Ganbold[3], Seungdo Jeong[4], Seonuck Paek[4], Yansen Su[5], Fan-Gang Tseng[2], and Tseren-Onolt Ishdorj[1,*]

[1]Department of Computer Science, Mongolian University of Science and Technology, Ulaanbaatar, Mongolia
[2]Department of Engineering and System Science, National Tsing Hua University, Taipei, Taiwan ROC
[3]Department of Physics, National University of Mongolia, Ulaanbaatar, Mongolia
[4]Department of Smart Information and Telecommunication Engineering, Sangmyung University, Chungnam, Republic of Korea
[5]School of Artificial Intelligence, Anhui University, Hefei, 230601, China
[6]Department of Computer Science, New Mongol Institute of Technology, Ulaanbaatar, Mongolia
[*]tseren-onolt@must.edu.mn

The datasets generated during the current study are available in the GitHub repository. Training and testing were conducted using an NVIDIA RTX 3090 GPU.

Model	Accuracy		Precision		Sensitivity		Specificity		f1-score	
	real data	augm. data	real data	augm. data	real data	augm. data	real data	augm. data	real data	augm. data
RF	0.9231	0.9733	0.9524	0.9885	0.9524	0.9663	0.8000	0.9836	0.9524	0.9773
GBM	0.8846	0.9733	0.8750	0.9775	1.0000	0.9775	0.4000	0.9672	0.7000	0.9775
SVM	0.8462	0.9867	0.8696	1.0000	0.9524	0.9775	0.4000	1.0000	0.9091	0.9886
KNN	0.8846	0.9800	0.9500	0.9886	0.9048	0.9775	0.8000	0.9836	0.9268	0.9831
DeepATsers	0.8462	1.0000	0.8696	1.0000	0.9524	1.0000	0.4000	1.0000	0.9091	1.0000

Supplementary Table S1: ML and DL model binary classification analysis of SARS-CoV-2 proteins with and without augmented experimental datasets, respectively. DeepATsers outperforms ML models on augmented datasets for the most measurements.

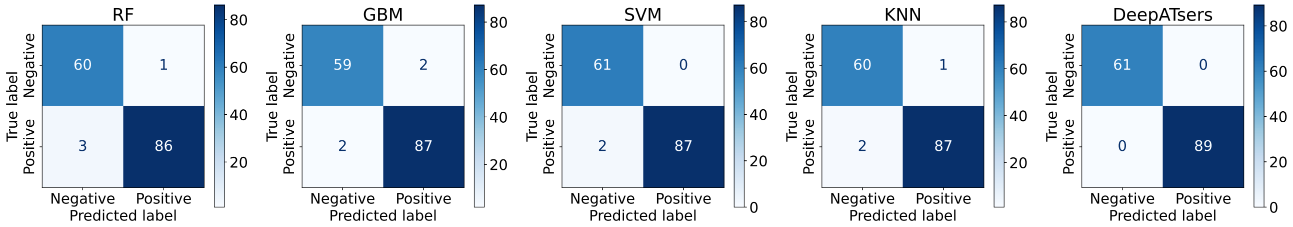

Supplementary Figure S1: Confusion matrices of ML and DL models for binary classification of SARS-CoV-2 proteins on the augmented spectral dataset.

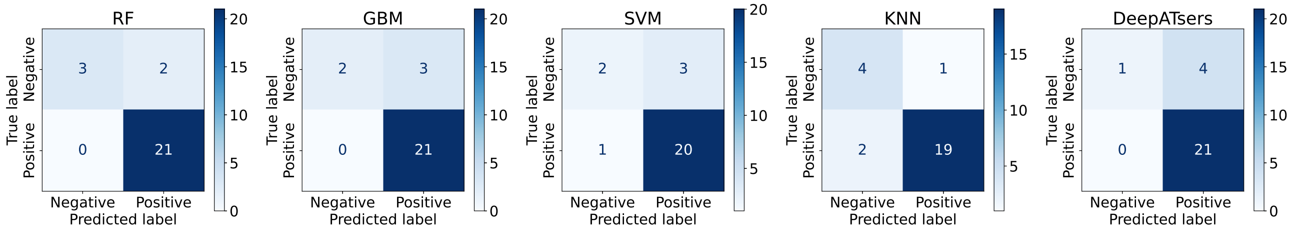

Supplementary Figure S2: Confusion matrices of ML and DL models for binary classification of SARS-CoV-2 proteins on the real spectral dataset.

Model	Accuracy		Precision		Sensitivity		Specificity		f1-score	
	real data	augm. data	real data	augm. data	real data	augm. data	real data	augm. data	real data	augm. data
RF	0.5600	0.9437	0.4933	0.9465	0.5600	0.9437	0.7904	0.9851	0.5040	0.9432
GBM	0.5200	0.9500	0.3404	0.9551	0.5200	0.9500	0.7904	0.9872	0.4095	0.9505
SVM	0.6000	0.9375	0.4745	0.9409	0.6000	0.9375	0.8281	0.9838	0.5268	0.9380
KNN	0.6400	0.9187	0.5806	0.9245	0.6400	0.9187	0.8520	0.9781	0.6038	0.9177
DeepATsers	0.6000	0.9750	0.5529	0.9757	0.6000	0.9750	0.7594	0.9984	0.5479	0.9750

Supplementary Table S2: ML and DL multi-class classification results for SARS-CoV-2 proteins using the real experimental dataset and the GAN augmented dataset, respectively.

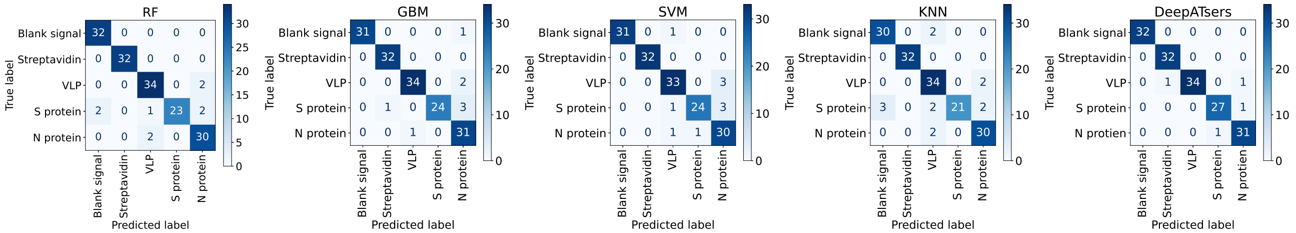

Supplementary Figure S3: Confusion matrices for multi-class classification of SARS-CoV-2 proteins on the augmented spectral dataset.

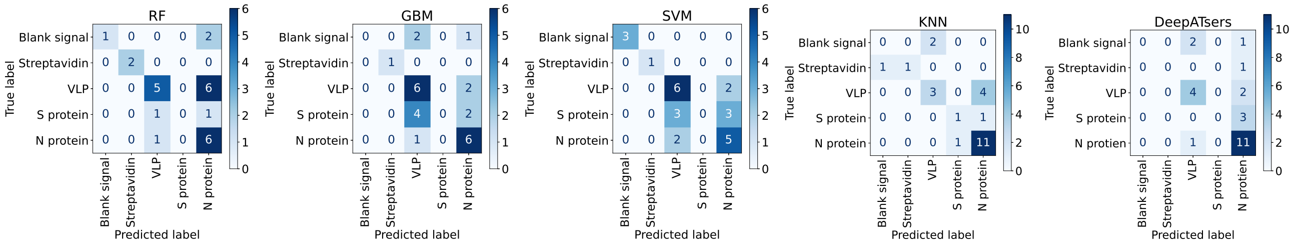

Supplementary Figure S4: Confusion matrices for multi-class classification of SARS-CoV-2 proteins on real spectral dataset.

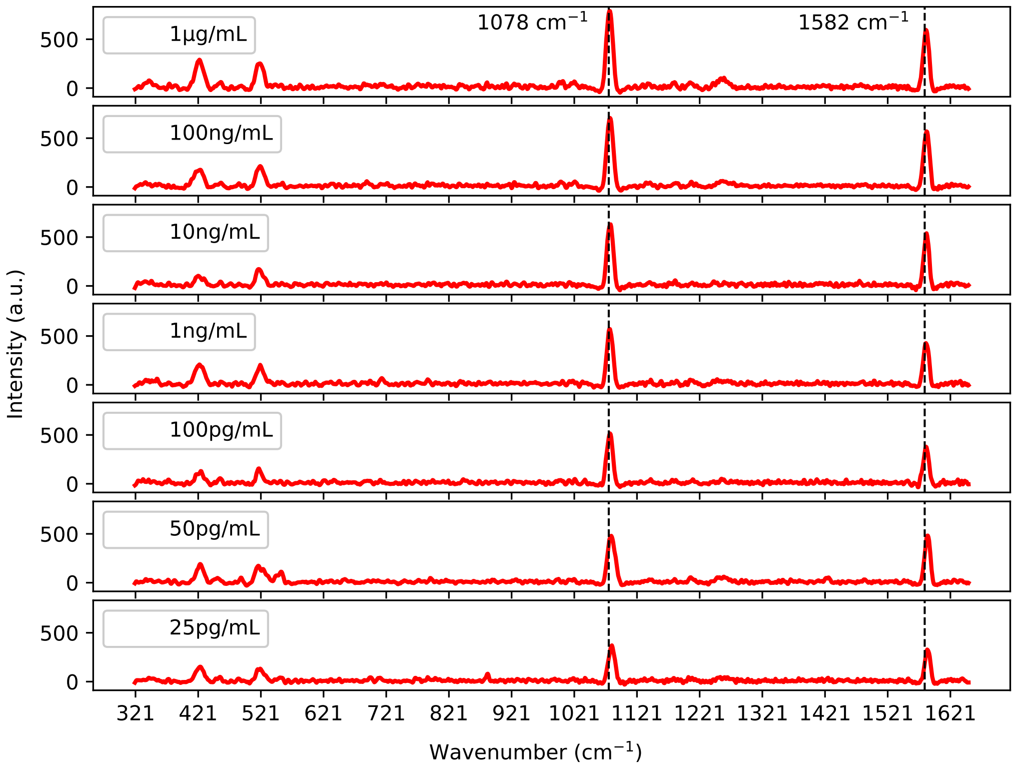

Supplementary Figure S5: Independent SERS spectra of SARS-CoV-2 antigen from seven different concentrations of Omicron variant.

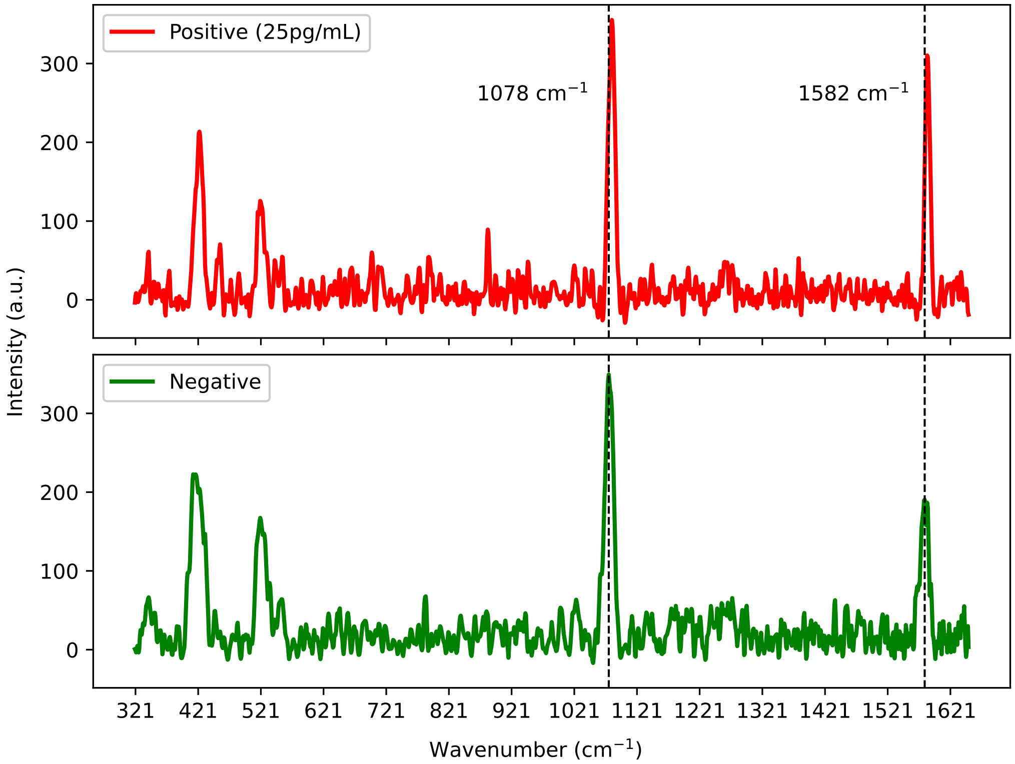

Supplementary Figure S6: SERS spectrum of SARS-CoV-2 Omicron variant with the lowest concentration (25pg/mL) (above) vs SERS spectra of the negative samples (below). Two samples had quite similar characteristics in Raman signal intensities.

Hyperparameters	Value	Optimal Value
Batch size	[10, 32, 40]	10
Epochs	[50, 100, 120]	50
Optimizer	['SGD', 'Adam', 'RMSprop']	Adam
Activation	['softmax', 'tanh', 'relu']	ReLu
Learn rate	[0.001, 0.01]	0.01
Dropout rate	[0.2, 0.4, 0.6]	0.2

Supplementary Table S3: Hyperparameter Grid Search Space and Suggested Values.

To set up optimal hyperparameters in the DeepATsers model, an automatic parameter tuning algorithm, Grid search, was employed. These parameters are presented in Supplementary Table S3. Then the hyperparameters were applied to binary classification and resulted with high accuracy as shown in Supplementary Figure S7. However, multi-class classification using the same hyperparameters was not good enough, having only an accuracy of 0.67. See Supplementary Figure S8.

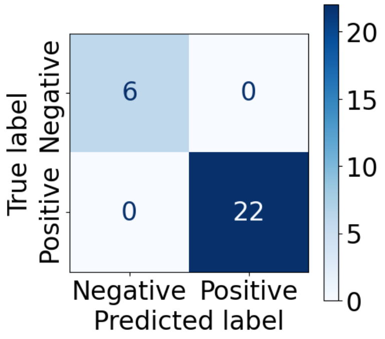

Supplementary Figure S7: Binary classification of Omicron variant detection using the hyperparameters obtained by Grid search presented in Supplementary Table S3.

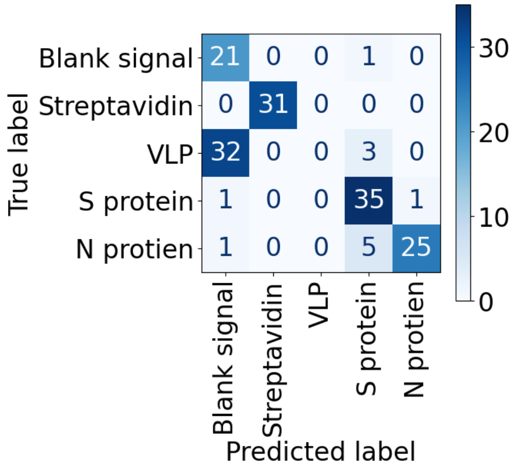

Supplementary Figure S8: Multi-class classification of SARS-CoV-2 proteins applying the same hyperparameters used in Supplementary Figure S7.
